# BIOFILM: FORMATION AND NATURAL PRODUCTS’ APPROACH TO CONTROL – A REVIEW

**DOI:** 10.21010/Ajid.v16i2S.7

**Published:** 2022-08-17

**Authors:** Osungunna Michael Oluwole

**Affiliations:** Department of Pharmaceutical Microbiology, Faculty of Pharmacy, Obafemi Awolowo University, Ile-Ife, Osun State, Nigeria

**Keywords:** Biofilm, formation, natural products, approach, control

## Abstract

Biofilm formation, especially on indwelling medical devices such as catheters, can result in infections and substantially affect patients’ quality of life. Biofilm-associated infections have led to increased morbidity and mortality, increased cost of treatment, and length of hospital stay. However, all of the identified consequences of the biofilm-associated infections had been attributed to the reduced susceptibility of biofilm to conventional antimicrobial agents which has necessitated the development of a new strategy for biofilm infections control, thereby making a search for more effective antimicrobial agents from plant source inevitable. So far, some antimicrobial agents (crude or isolated compounds) from plant sources affect a specific stage of biofilm development while a few of them have been developed into a suitable dosage form for biofilm control. In this review, an attempt is made to look into some definitions of biofilm by “biofilmologists”, stages in biofilm formation, mechanisms of resistance in biofilm, biofilm control strategies, the use of some natural products in biofilm control and concepts of probiotics as agents of biofilm control.

## Introduction

There is no single universal definition of biofilm. There are many definitions of biofilm as there are “biofilmologists”. However, despite an avalanche of biofilm’s definitions, Dolan and Costerton (2002) gave an all-encompassing definition of biofilm as “a microbially derived sessile community characterized by cells that are irreversibly attached to a substratum or interface or each other, are embedded in a matrix of extracellular polymeric substances that they have produced, and exhibit an altered phenotype concerning growth rate and gene transcription”.

The autogenic extracellular polymeric substances (EPS), although composite with respect to the biofilm-forming bacteria, perform the same roles which include dissemination of necessary nutrients for cellular growth (Cheng *et al.*, 2007), trapping of external nutrients for cellular sustenance (Cheng *et al.*, 2007) and protection from accidental stress as opposed to planktonic bacteria (Pang *et al.*, 2005). Extracellular polysaccharides, proteins and DNA are the major components of EPS. Also, some bacteria produce other substances which make their matrix unique. For instance, cellulose has been reported as a crucial component of the extracellular matrix of *Salmonella typhimurium* and *Escherichia coli* (Zogaj *et al.*, 2001; Solano *et al.*, 2002). The extracellular polysaccharides can be capsular or exopolysaccharides. The polysaccharides that remain associated with cell following cellular harvest and centrifugation is capsular while those in the supernatant are exopolysaccharides (Branda *et al.*, 2005). Myriad benefits that have been ascribed to the polysaccharide component of the biofilm matrix include adhesion, protection, and structure (Limoli *et al.*, 2015).

On the other hand, the protein content of the biofilm matrix plays diverse functions in the formation of biofilm and its dissolution. They are involved in the attachment of biofilm cells to surfaces, stabilization of biofilm matrix via interactions with the components of exopolysaccharide and nucleic acid, development of three-dimensional biofilm configurations, and dissolution of biofilm matrix through enzymatic degradation of polysaccharides, proteins, and nucleic acids (Fong and Yildiz, 2015). Also, the role played by the extracellular DNA in the initiation and eventual formation of biofilm structure cannot be underscored (Whitchurch *et al.*, 2002). Despite seeming similarity in some components of biofilm matrix, it worths note that developed biofilms are not structurally homogeneous monolayers of microbial cells on a surface but rather heterogeneous in both time and space (Lewandowski, 2000). Biofilm formation offers protection against antibiotics (Goldberg, 2002), disinfectants (Peng *et al.*, 2002) and dynamic environment (Chen *et al.*, 1998) of which protection from environmental insults and assaults is foremost (Davey and O’Toole, 2002).

### Formation of biofilm

**Figure 1:**
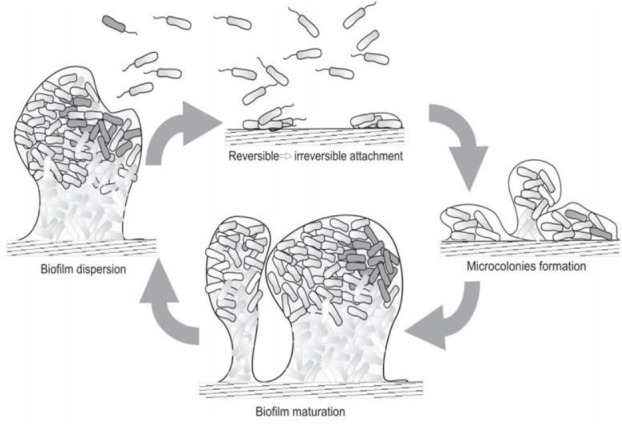
Stages involved in the formation of biofilm

Formation of biofilm requires the presence and interaction of microbes and surface (biotic or abiotic). The stages involved in the formation of biofilm are exemplified in Figure 1.

The earliest stage in the formation of biofilm is an attachment which can be reversible or irreversible. The reversible attachment, also known as the docking phase, is governed by some physicochemical parameters that characterize the association between the two interacting surfaces of the bacterial cell and the surface of choice that has been conditioned for the purpose (An *et al.*, 2000). Docking is determined by the net addition of forces (attractive or repulsive) produced when the organism is brought close to the surface for attachment, with organism being propelled randomly or by way of chemotaxis and motility (Dunne, 2002). Such forces (attractive or repulsive) include hydrophobic and electrostatic interactions, van der Waals forces, temperature, steric hindrance, and hydrodynamic interactions. Because majority of bacteria and static (inert) surfaces are usually negatively charged, electrostatic attractions tend to favour repulsion. The situation, however, differs where organism assumes overall positive charge at prevailing pH, thereby promoting docking to negatively charged substrates e. g. Teflon. Hydrophobic associations seem to have a much impact on the aftermath of the docking phase (Carpentier and Cerf, 1993). It has been reported that the more hydrophobic cells attach more strongly to hydrophobic surfaces, such as *E. coli* and catheter, while hydrophilic cells strongly attach to hydrophilic surfaces, such as *S. aureus* and glass surface (Giaouris *et al.*, 2009). Reversible attachment is usually followed by irreversible attachment, often called locking phase, and it is usually molecularly mediated using cell-produced adhesin.

This stage is made possible because bacteria physical appendages (flagella, fimbriae and pili) have been able to overcome the repulsive physical forces of the electrical double layer (De Weger *et al.*, 1987). The defeat of repulsive physical forces makes contact with the bulk lattice of the conditioning layer stimulate chemical reactions such as oxidation and hydration (Ganesh and Anand, 1998) thereby consolidating the bacteria-surface bond. At this stage, adhesion process becomes consolidated by weakly-attached organisms through the production of exopolysaccharides that bond with substrates and ligands that are receptor-specific found on fimbriae, fibrillae, and pili, or both. After this stage, the organism becomes firmly attached to the surface because when physical or chemical intervention is lacking, adhesion becomes permanent and irreversible (Dunne, 2002). Attachment process can be affected by many interrelated factors such as the roughness and smoothness of the surface, characteristic nature of the aqueous medium including the temperature, pH, ionic strength and nutrient levels as well as the cell surface properties like possession of flagella/fimbriae, hydrophobicity, and EPS production (Donlan, 2002).

Biofilm growth and maturation begins following irreversible attachment of bacteria to the surface. Biofilm maturation is characterized by overall increase in density and complexity of biofilm due to active replication of surface-bound bacteria, interaction between organic and inorganic particles in the immediate vicinity and the extracellular components generated to create EPS and domination over physical and chemical contribution to attachment by a biological process. However, the growth potential of any bacterial biofilm is limited by factors as the waste removal and availability of nutrients in the immediate environment. Nonetheless, there is an ideal hydrodynamic flow which favours growth and perfusion in preference to gradual removal of the external layers (Carpentier and Cerf, 1993). Osmolarity, internal pH, oxygen perfusion, and carbon source are other factors controlling biofilm maturation (O’Toole and Kolter, 1998). The corollary to this submission is that biofilm structure can be affected by those factors that control biofilm maturation. Initially, biofilms were represented as simple homogenous planar structure, largely 2D, with a relatively constant thickness. With new technologies, however, serious doubt had been thrown on the homogenous flat structure proposition. To date, three schools of thought exist on biofilm structure which include the homogeneous model, the heterogeneous mosaic model and the mushroom or tulip model (Petrova and Sauer, 2016).

Biofilm growth and maturation continues until a critical mass is attained where a dynamic equilibrium starts to give rise to planktonic organisms from the external layer of growth, being the farthest from the surface. The organisms generated can break free from the biofilm and populate other surfaces (Wimpenny *et al.*, 2000). The escape is the beginning of the biofilm dispersal stage which has been described as the start of a new biofilm cycle rather than the final stage of biofilm formation. It is a sophisticated process that involves several environmental signals such as the availability of nutrients and quorum sensing, among others (Karatan and Watnick, 2009) which aids microbial survival, biological dispersal and disease transmission (Kaplan, 2010). The environmental signals can be mechanical as expressed by *B. subtilis* where an inverse relationship between motility and surface attachment has been reported. *Also, e*nvironmental glucose and catabolite repression have been reported to impede the formation of multilayer biofilm in a number of clinical and laboratory strains of *E. coli*, a variety of clinical isolates of *Enterobacteriaceae*, including *B. subtilis*. Low concentration of glucose (0.1%) activated *B. subtilis* biofilm formation while high concentration of glucose inhibited its biofilm formation. Moreover, while Gram-negative bacteria use acylated homoserine lactone (AHL) as autoinducers to regulate their gene expression in a manner that is dependent on cell density, Gram-positive bacteria use a modified oligopeptide as autoinducers (Lawrence *et al.*, 2002). The dispersal stage in biofilm can be classified into (i) disengagement of cells from biofilm community; (ii) transfer of detached cells to a fresh territory; and (iii) attachment of detached and transfered cells to a surface in the fresh territory. The 3 phases can be generated actively by the bacteria themselves or passively by outer forces such as predator grazing, fluid shear, human intervention and abrasion (collision between solid particles and the biofilm) (Ymele-Leki and Ross, 2007; Karatan and Watnick, 2009). Dispersal of biofilm can be achieved under three different modes which include: erosion (the continuous and low level release of isolated or minute clusters of cells from a biofilm during the process of biofilm formation), sloughing (the instantaneous detachment of large portions of the biofilm, usually during the later stages of biofilm formation) (Stoodley *et al.*, 2001; Wilson *et al.*, 2004), and seeding/central hollowing (release of a single or clusters of cells from inside the biofilm community) (Boles *et al.*, 2005; Ma *et al.*, 2009). However, while sloughing and abrasion can be achieved either actively or passively, seeding is often achieved actively (Kaplan, 2010). It is, however, important to note the following as regards the biofilm dispersal stage that (i) Cells closest to the surface are characterized by reduced growth and metabolic activities due to absence of nutrients, reduced pH, pO_2_, or an accumulation of harmful metabolites (Prosser *et al.*, 1987); (ii) Cell-cell signalling compounds, such as acylated homoserine lactones, which control the gene expression based on population density may regulate the processes of initial formation, maturation, and dispersal of a biofilm (Davies *et al.*, 1998); (iii) once fully matured, a form of functional general harmonization and control that imitates primordial eukaryotic tissue can be generated by biofilm through alteration in the patterns of bacterial growth, physiological cooperation, and efficient metabolism (Costerton *et al.*, 1987); and (iv) Different bacteria utilize different mechanism of biofilm dispersal (Kaplan, 2010).

### Resistance in biofilms

The same strain of bacteria when grown as planktonic and sessile (biofilm) will exhibit variation in their susceptibilities hence resistance to the same antibacterial agent (Anderl *et al.*, 2000). This is an indication that the mechanisms of antibacterial resistance in planktonic bacterial cells and biofilms differ. Also, host factors are not required for the manifestation of biofilm resistance to antibiotics thereby allows for reproducibility of antibiotic resistance *in vitro* (Stewart, 2002). The existence of genes coding for multiple antibacterial resistance in biofilms has no important role in mediating biofilm resistance to an antibacterial agent (Brooun *et al.*, 2000; Maira-Litran *et al.*, 2000). This invalidates the possibility of resistance acquisition by biofilms through mutations or mobile genetic elements (Anwar *et al.*, 1989; Williams *et al.*, 1997). Susceptibility to antibiotics is usually rapidly restored following bacterial cells dispersion from a biofilm, to underscore an adaptive resistance mechanism rather than a genetic alteration (Stewart, 2002). However, biofilms treated repeatedly or for a long time can develop conventional antibacterial resistance (Bagge *et al.*, 2000). It, therefore, follows that one should look beyond conventional mechanisms of resistance to understand biofilms resistance. Several hypotheses have been proposed for a proper understanding of mechanisms of resistance in a biofilm.

The hypothesis about limited antibiotic penetration implies that only the exterior of a biofilm is exposed to a lethal concentration of the antibiotic attributed to a diffusion barrier that regulates the movement of the antibiotic into the bacterial biofilm. There are reservations to this hypothesis as some authors have reported retarded penetration of antibiotics (Kumon *et al.*, 1994; Shigeta *et al.*, 1997) into the biofilm matrix, while others indicate rapid penetration (Suci *et al.*, 1994). The ability of antibiotics to penetrate biofilm has therefore been attributed to its type. For instance, Penicillin class of antibiotics can penetrate biofilm formed by β-lactamase negative strains of bacteria better than biofilm formed by β-lactamase positive strains of the same bacteria due to the ability of the later to deactivate penicillin (Anderl *et al.*, 2000). Also, aminoglycosides antibiotics become adsorbed unto the matrix of biofilm due to its interaction (being positively charged) with negatively charged polymers in the biofilm matrix, thereby retarding their penetration (Kumon *et al.*, 1994; Shigeta *et al.*, 1997).

The physiological limitation hypothesis of reduced susceptibility of biofilms to antibiotics implies that some microbial cells within the biofilm exist in a phenotypic state that is more recalcitrant. This hypothesis takes into consideration the effects of factors such as the rate of growth, the age of the biofilm and starvation on biofilm susceptibility to antibiotics. For instance, Aires *et al*. (2017) reported reduced susceptibility of *K. pneumoniae* biofilm to Ceftriaxone, a cephalosporin antibiotic, with increasing age of the biofilm in a similar way Singla *et al*. (2013) reported reduced susceptibility of *K. pneumoniae* to ciprofloxacin, amikacin and piperacillin antibiotics with an increase in age of the biofilm. The physiological complexity of biofilm increases with age hence reduced susceptibility. Notwithstanding this submission, there have been reports of newly formed biofilms being resistant despite being too narrow to pose an impediment to the penetration of an antimicrobial agent or metabolic substrates (Das et al., 1998; Cochran *et al.*, 2000). This has made this hypothesis speculative.

Another hypothesis to explain the reduced antimicrobial sensitivity of biofilms is the variation in chemical microenvironment within the biofilm. Bacteria at the surface of biofilm have access to oxygen more than those at the interior. This may result in the formation of two distinct environments inside the biofilm with an aerobic environment at the surface and anaerobic in the interior (de Beer *et al.*, 1994). The same explanation holds for nutrients and such other growth conditions. The corollary to this submission is reduced growth rate and metabolism on the inside in contrast to the exterior of the biofilm. Because of the high metabolism at the biofilm surface, there may be an accumulation of waste products such that the pH of the surrounding changes compared to what obtains at the biofilm interior (Zhang and Bishop, 1996). All these variations may lead to reduced activity of antibiotics on biofilm. For instance, aminoglycoside antibiotics are more effective in an aerobic environment than in anaerobic environment (Tack and Sabath, 1985). This implies that bacteria at the surface of biofilm will be killed at the expense of those at the interior. Penicillin acts on actively growing (actively metabolizing) cells because the target is cell-wall synthesis (Tuomanen *et al.*, 1986), such that bacteria at the biofilm surface will be killed by penicillin while less metabolizing ones at the interior will be spared.

The hypothesis that bacteria in biofilm behave like spores remains a strong and all-inclusive explanation of reduced susceptibility of biofilms to antibiotics (Stewart and Costerton, 2001).

It is important to note that bacteria in biofilm may display any of the mechanisms of resistance alone or in combination regardless of the clinical site of infection as shown in Tables 1 and 2 below (Simoes *et al.*, 2010):

**Table 1:** Biofilm-related bacterial infections

Associated microbe	Biofilm-related infection
*Escherichia coli*	catheter-associated urinary tract infection, acute and recurrent urinary tract infection, biliary tract infection
*Pseudomonas aeruginosa*	catheter-associated urinary tract infection, cystic fibrosis lung infection, contact lens-related keratitis, persistent wound infection, recurrent rhinosinusitis, recurrent otitis media
*Staphylococcus aureus*	recurrent rhinosinusitis, persistent osteomyelitis, otitis media, endocarditis, long-term orthopaedic implants
*Streptococcus pneumoniae*	recurrent rhinosinusitis, colonization of nasopharynx, long-term obstructive pulmonary disease, recurrent otitis media.
*Streptococcus pyogenes*	Colonization of oral cavity and nasopharynx, recurrent tonsillitis

**Table 2:** Selected medical devices and associated biofilm-forming bacteria

Medical devices	Bacterial species
Artificial hip prosthesis	*Bacteroides species, β-haemolytic bacteria, Coagulase- negative Staphylococci,Enterococci,Escherichia coli, Proteus mirabilis,Staphylococcus aureus*
Artificial voice prosthesis	*Candida albicans, Candida tropicalis, Staphylococcus epidermidis, Stomatococcus mucilaginous, Streptococcus mitis, Streptococcus salivarius, Streptococcus sobrinus*
Central venous catheter	*Candida albicans, Coagulase-negative Staphylococci, Enterococcus faecalis, Klebsiella pneumoniae Pseudomonas aeruginosa, Staphylococcus aureus*
Intra-uterine device	*Candida albicans, Corneybacterium species, Enterococcus species, group B Streptococcus, Lactobacillus plantarum, Micrococcus species, Staphylococcus epidermidis, Staphylococcus aureus*
Prosthetic heart valve	*Coagulase-negative Staphylococci, Enterococci, Staphylococcus aureus, Streptococcus, Viridans*
Urinary catheter	*Enterococcus faecalis, Escherichia coli, Klebsiella pneumoniae, Proteus mirabilis, Staphylococcus epidermidis*

### Biofilm control Strategies

Control strategies for biofilms can be preventive or curative. While preventive strategies target the prevention of biofilm formation, curative measures target the formed mature biofilms.

Preventive measures include (i) stoppage of bacteria attachment; (ii) stoppage of biofilm growth; (iii) blockage of biofilm matrix synthesis; and (iv) disruption of cell-cell communication. Curative measures, on the other hand, include (i) killing of preform biofilm; (ii) promotion of preform biofilm detachment; and (iii) binding and elimination of preform biofilm. In practice, preventing biofilm formation would be a better option than treating it (Francolini and Donelli, 2010). However, to date, no known strategy can successfully manage the formation of unwanted biofilms without associated adverse side effects (Francolini and Donelli, 2010). The success of biofilm control depends on the knowledge of the mechanisms leading to biofilm formation (Sidhu *et al.*, 2001). However, both the preventive and curative measures can be achieved through the use of antimicrobial agents which can be conventional antibiotics and agents from a natural source, especially plants.

### Natural products and biofilm control

One of the ways by which biofilm can be controlled is through the use of antibiotics. However, due to poor and irrational antibiotics use which has made biofilm-related infections difficult to control, new biofilm control strategies are required (Banerjee *et al.*, 2019). Plants are known to represent a vast, sustainable resource of diverse classes of low molecular weight compounds with various biological activities. However, several plants and plant products have been evaluated for their anti-biofilm activity. The reported activities are evaluated based on any one of the mechanisms leading to biofilm formation.

### Anti-adhesion evaluation

The first stage in biofilm formation is the adhesion of bacteria to the surface. It, therefore, follows that agent that could prevent this step would be a potential antibiofilm agent. This is normally done by evaluating the capacity of the test organism to form biofilm when the agent being evaluated is present. The concentration is chosen such that it does not interfere with other antimicrobial activities such as growth inhibition and killing. As such, sub-inhibitory concentration is normally considered for antiadhesion evaluation. Many plant products have been evaluated for this activity towards the prevention of biofilm formation. A dose-dependent biofilm formation inhibition activity of methanolic extracts of *Sophora secundiflora, Sphaeralcea ambigua, Prosopis laevigata, Opuntia ficus-indica, Marrubium vulgare, Scutellaria drummondii, Nothoscordum bivalve* and *Gutierrezia microcephala* at subinhibitory concentrations against *E. coli* and *S. aureus* has been reported (Sánchez *et al.*, 2016). Similarly, biofilm formation inhibition of *Piper betle* against *S. mutans* ATCC 25175 and *A. actinomycetemcomitans* ATCC 33384 has been reported (Teanpaisan *et al.*, 2017). Sadansi *et al*. (2010) reported the ability of *Rosmarinus officinalis, Echinacea angustifolia, Thymus vulgaris* and *Mentha piperita* extracts to prevent attachment of *Listeria monocytogenes* ATCC 19111 by at least 50%. Other plants that have been evaluated for antiadhesion-based antibiofilm activity include *Agathosma betulina, Allium sativum, Aloe vera, Aspalathus linearis, Camellia sinensis, Glycyrrhiza glabra, Hypericum perforatum, Leptospermum petersonii, Melaleuca alternifolia, Syzygium aromaticum, Vaccinium macrocarpon* (van Wyk and Wink, 2004; Bozin *et al.*, 2007; Coetzee *et al.*, 2008).

### Cell-Cell communication Disruption

During biofilm formation, two types of interactions are possible: cell-substrate interaction (adhesion) and cell-cell interaction (cohesion) (Garrett *et al.*, 2008). The cell-cell interaction is usually in the form of signals mediated by the production of signal molecules called autoinducers (AIs). These signal molecules are only produced to a significant high local concentration at high cell density (Rutherford and Bassler, 2012) hence, the name “Quorum sensing”. Production of signal molecules leads to coordinated gene expression, which regulates various pathogenic processes such as biofilm formation (Bassler and Losick, 2006; Walters and Sperandio, 2006). It, therefore, follows that interference with this signal often leads to reduced pathogenicity (Rasmussen and Givskov, 2006). Targeting bacterial cell-cell signalling has allowed evaluating compounds without growth-inhibitory properties (Vickram *et al.*, 2011) and has been the focus of current research. Many plants and plant products have tested positive for interfering with quorum sensing thereby used in biofilm control. Vikram *et al*. (2010) reported flavonoids, particularly naringenin, quercetin, sinensetin, and apigenin from citrus, to regulate bacterial cell-cell interaction, formation of biofilm by *E. coli* O157: H7 as well as virulence in *V. harveyi*, with naringenin as a potent nonspecific inhibitor of cell-cell signaling mediated by autoinducer.

Similarly, Vikram *et al*. (2011) reported the ability of some purified limonoids from citrus to impede cell-cell interaction and formation of biofilm in *V. harveyi*. Ichangin, isolimonic acid, and deacetylnomilinic acid glucoside exhibited considerable inhibition of cell-cell signalling mediated by autoinducer and formation of biofilm. Emodin, one of the free anthraquinone compounds and a major active constituent in rhubarb (Ma *et al.*, 2008) has been reported to interfere with quorum sensing in *Ps. aeruginosa* and subsequent biofilm formation (Ding *et al.*, 2011). Other metabolites with anti-quorum sensing activity include furanone (Givskov *et al.*, 1996), L-canavanine (Keshavan *et al.*, 2005), cinnamaldehyde (Niu *et al.*, 2006), ajoene (Jakobsen *et al.*, 2012), Iberin (Jakobsen *et al.*, 2012), caffeine (Norizan *et al.*, 2013), curcumin (Packiavathy *et al.*, 2013), and carvacrol (Burt *et al.*, 2014). Anti-quorum sensing evaluation has not been restricted to isolated compounds only as crude extracts of some plants have also been evaluated (Damte *et al.*, 2013; Sarabhai *et al.*, 2013).

### Mature/Preformed biofilms

There are two possibilities when a mature biofilm is the target of biofilm control. It is either the mature biofilm is killed, or it is detached from the adhered surface. It has been reported that to kill biofilm requires about 100-1000 times the concentration required to kill planktonic cells (Sedlacek and Walker, 2007). One advantage of natural products is their safe status which may make high concentrations tolerable to host unlike conventional antibiotics (Rodrigues *et al.*, 2012). The rate of kill of biofilm as a function of age with young biofilms being more susceptible to eradication by antimicrobials than old biofilms has also been reported (Aires *et al.*, 2017).

However, when mature biofilm is the target for control, all factors that play roles in reduced susceptibility of biofilm to antimicrobials must be considered. It is worth noting that agents may have excellent antiadhesion property but may fail to affect preformed biofilms. For instance, while *Mentha piperita* extract showed excellent antiadhesion property against *Pseudomonas aeruginosa* biofilm formation, it only showed 38% inhibition of preformed biofilm of the same organism. Similarly, *M. piperita* and *Aspalathus linearis* showed 28% and 8% inhibition of preformed biofilm of *Candida albicans*, respectively. However, there was enhanced biofilm development observed for *Thymus vulgaris*, *Rosmarinus officinalis*, *Melaleuca alternifolia* and *Echinacea angustifólia* against the biofilm of both organisms (Sandasi *et al.*, 2011).

### Other natural products

### Honey

Honey has been utilized in medicine for centuries by Chinese, Assyrians, Eagyptians and Greeks to treat wounds and manage diseases (Sandasi *et al.*, 2011). Studies have revealed that honey prevents the adherence of *Salmonella enteritidis* to isolated intestinal epithelial cells (Alnaqdy, 2005). However, the ability of honey to inhibit formation of biofilm, cause biofilm detachment as well as kill preformed biofilms of *Proteus mirabilis*, and *Enterobacter cloacae* has been reported (Majtan *et al.*, 2013). In the report, biofilm development of both isolates was significantly reduced by all honey samples evaluated at 10% (w/v) sub-inhibitory concentration. Likewise, at 50% (w/v) concentration, a significant partial detachment of *P. mirabilis* biofilm after 24 h was caused by each of the honey samples evaluated while no honey sample was able to cause a significant detachment of *E. cloacae* biofilm. Additionally, *P. mirabilis* and *E. cloacae* biofilms treatment with all honey samples brought about 0.35–1.16 and 1.2–7.5 log units significant decrease in biofilm counts per well, respectively.

### Cranberries

Cranberries have long been the focus of interest for their beneficial effects in preventing urinary tract infections (Harkins, 2000; Vatem *et al.*, 2005). The benefits derived from the use of cranberry juice may be related to its ability to inhibit bacterial adherence (Sobota, 1984). The A-type oligomeric proanthocyanidins (condensed tannin), which make up around 65 percent of the non-dialyzable substance in cranberries, are the most active component (NDM) (Bodet *et al.*, 2006). Cranberry A-type proanthocyanidins have been reported to have activity against *Escherichia coli* biofilm (Foo *et al.*, 2000; Howell *et al.*, 2005). However, proanthocyanidins have been tested against *Pseudomonas aeruginosa* biofilms for their capacity to reduce biofilm formation, biofilm detachment, and biofilm growth (Ulrey *et al.*, 2014).

According to the study, cranberry proanthocyanidins (PACs) effectively impeded the formation of *P. aeruginosa* biofilm *in vitro* in a concentration-controlled manner with 40.9 and 55.7 percentage biofilm inhibition at 1 and 10 µg/mL concentrations, respectively. However, 10 and 100 µg/mL PACs exhibited 54.1 and 39.6 percent reduction of preformed *P. aeruginosa* biofilm. PAC therapy (*in vivo*), on the other hand, had no effect on *P. aeruginosa* attachment.

### The concept of probiotics as potential antibiofilm agents

Probiotic was basically coined to describe substances produced by one microbe which promote the growth of other microorganisms, but it has since been expanded to include animal feed supplements that help animals’ intestinal flora balance as well as tissue extracts that promote microbial growth (Fuller, 1999). Probiotic comes from the Greek language and means “for life.” However, as passion for the utilization of live bacterial supplements has grown, so is the knowledge of their mechanisms of action. This has broadened the definition of probiotics. The definition that “probiotics are live microbial feed supplements that benefit the host animal by increasing microbial balance,” is the most generally used and has made a significant contribution to the development of the notion of probiotics” (Stewart and Costerton, 2001).

Probiotics are currently defined as “live microorganisms that bestow a health benefit on the host when provided in suitable levels” by the Food and Agriculture Organization of the United Nations World Health Organization. However, the health advantages may not appear until probiotics are ingested in sufficient quantities as part of a balanced diet” (FAO/WHO, 2001). Although there is a lack of data on the minimum effective dose, it is widely accepted that probiotic products should contain at least one million (10^6^) bacterial counts per mL or gram of probiotic microorganisms and that a total of one hundred million (10^8^) to one billion (10^9^) probiotic microorganisms should be ingested daily for the manifestation of probiotic effect in the consumer. Other conditions for a probiotic to provide benefits include (i) acid and bile tolerance (ii) adhesion to mucosal and epithelial surfaces, (iii) activity against pathogenic bacteria, and (iv) bile salt hydrolase activity (Kechagia *et al.*, 2013). The strains must also be able to proliferate in the manufacturing environment and maintain viability in typical storage settings (Toma and Pokrotnieks, 2006; Sanders, 2008).

Despite these requirements, it’s worth noting that no precise parameters are required for all probiotic uses, and the ideal way to determine the qualities of a strain is to conduct target demographic as well as the target physiologic function-specific investigations (Saarela *et al.*, 2000; FAO/WHO, 2002; Mercenier, Lenoir-Wijnkoop and Sanders, 2008). Although there are many microbial species capable of exerting probiotic qualities in terms of definition, those belonging to the genera Lactobacillus and Bifidobacterium have been considered significant and useful (Holzapfel *et al.*, 2001; Mercenier *et al.*, 2003; Singh *et al.*, 2013). Probiotics will differ in their modes of action, which range from bacteriocin and short-chain fatty acid synthesis, gut pH reduction, and nutrition competition to mucosal barrier function stimulation and immunomodulation (Kechagia *et al.*, 2013). Lactobacillus strains, for example, have been shown to exhibit antibacterial properties against enteric bacterial infections (Lievin-Le *et al.*, 2014). The ability of *Lactobacillus casei* to produce bacteriocins and biosurfactant proteins with antiadhesion property against *S. aureus*, *B. subtilis*, and *Micrococcus roseus* has been reported (Golek *et al.*, 2009; Sharma and Singh, 2014). Reuterin, a broad-spectrum antimicrobial produced by *Lactobacillus reuteri* in the human gastrointestinal tract, is effective against broad spectrum enteric infections (Spinler *et al.*, 2008). Lactobacilli, probiotic bacteria, can also reduce the virulence and spread of pathogenic diseases (Servin, 2004) through the production of bacteriocins, lipopeptides, and surface proteins (Sharma and Singh, 2014; Satpute *et al.*, 2016).

However, the ability of probiotics to prevent biofilm formation and growth has been reported. Taheur *et al*. (2016), for example, investigated the antibacterial and antibiofilm properties of Lactic acid bacteria derived from barley, fermented olive and conventional dried meat against oral infections. The findings demonstrated that *Lactobacillus brevis*, one of the isolated bacteria, can prevent *Bacillus cereus* ATCC 14579 and *Streptococcus salivarius* B468 from forming biofilms. The study found, however, that the *Lactobacillus brevis* might be used as probiotics in functional foods and, in particular, in the management of oral infections. The applicability of *L. kefiranofaciens* species designated as DD2, DD5, and DD6 obtained from kefir as well as *L. johnsonii* JCM 1022, *L. plantarum* ATCC 10012, and *L. rhamnosus* ATCC 7469 as possible probiotics that can be administered orally with reference to their antimicrobial activity, ability to survive in a practical oral condition, and anti-biofilm efficacy against *Streptococcus mutans* and *Streptococcus sobrinus* has been investigated (Jeong *et al.*, 2018). The results revealed that strains DD2, ATCC 10012, ATCC 7469, and JCM 1022 showed strong aerotolerance and enzymatic resistance as indices of optimal oral survivorship, and reduced the development and growth of *S. sobrinus* and *S. mutans* biofilms. DD2 repressed all the biofilm-forming genes involved in glucose metabolism, as well as those that encode regulatory biofilm and adhesion proteins. According to the findings, *L. kefiranofaciens* DD2 inhibits *S. sobrinus and S. mutans*, the two major causal bacterial pathogens of dental caries, effectively and directly. Wasfi *et al*. (2018) evaluated the antibacterial and antibiofilm properties of *Lactobacillus* sp. (*Lactobacillus casei*, ATCC 393; *Lactobacillus reuteri*, ATCC 23272; *Lactobacillus plantarum*, ATCC 14917, and *Lactobacillus salivarius*, ATCC 11741) against *S. mutans* (ATCC 25175). *Lactobacillus salivarius* supernatant was found to reduce *S. mutans* adhesion and preformed biofilm by 87 percent and 47 percent, respectively, with a significant reduction (P< 0.01). The effect of *L. casei* supernatant on adhesion was the smallest of the studied supernatants, with no influence on the preformed biofilm. The supernatant of *L. plantarum* and *L. reuteri* induced a reduction in adhesion of 81.7–80.5 percent and a reduction in preformed biofilm of 26.5–24.7 percent. *Lactobacillus* sp., according to the findings, can limit tooth decay and regulate dental caries by reducing cell adhesion and preformed biofilms, among other things.

Nonetheless, the antibiofilm activity of probiotics against persistent infections had been reported. *Lactobacillus rhamnosus* and *Lactobacillus gasseri* strains were found to have antimicrobial and antibiofilm activity against three biofilm-forming pathogens commonly involved in chronic infections, namely: *E. coli*, *Pseudomonas aeruginosa*, and *Staphylococcus aureus*, according to Osama *et al*. (2017). Similarly, there had been a report of a strong bactericidal and antibiofilm activity of cell-free supernatant produced by standard and commercial probiotic strains (*L. acidophilus* LA14, *L. acidophilus* ATCC 4356 and *L. rhamnosus* ATCC 9595) against Multi-Drug-Resistant *E. coli* isolated from fish fillet (Fernandes, 2019). Hager *et al*. (2019) evaluated the ability of a novel probiotic formulation of *Lactobacillus rhamnosus*, *Saccharomyces boulardii*, *Bifidobacterium breve*, and *Lactobacillus acidophilus* supplemented with amylase as a hydrolytic enzyme, to manage polymicrobial biofilms formed by a combination of *E. coli, C. tropicalis* and *S. marcescens*. The work concluded that the formulated probiotic might have capacity to disrupt formation of polymicrobial biofilm thereby useful in the management of inflammatory bowel diseases.

Probiotic Lactobacilli were tested for antibacterial, antibiofilm, and antiadherence effects against a multidrug-resistant *Proteus mirabilis* derived from urine samples by Shaaban *et al*. (2020). In the study, the possibility of using *Lactobacillus casei* and *Lactobacillus reuteri* in the treatment of Proteus-associated urinary tract infections was reported due to the antibacterial and antibiofilm properties exhibited by both the cultures and cell-free supernatants of the 2 Lactobacilli species.

## Conclusion

Despite a large number of plants and plant products that have been reported for their antibiofilm activity, few, if any, have been transformed into dosage forms thereby making reliance on conventional antibiotics inevitable despite reported reduced susceptibility of biofilms to conventional antibiotics associated with a high incidence of biofilm-related infections worldwide. Efforts should be directed at formulating these plant products to make them available for use to control biofilms-associated infections, taking into cognizance the various stages of biofilm development.

### Declaration of Conflict of Interest

There is no conflict of interest associated with this work.

**List of Abbreviations:**
ATCC: American Type Culture Collection,
DNA: Deoxyribonucleic Acid,
mEPS: Extracellular Polymeric Substance,
FAO: Food and Agriculture Organization,
JCM: Japan Collection of Microorganisms
NDM: Non-Dialyzable Materials,
WHO: World Health Organization,
PACs: Proanthocyanidins
